# LexicHash: sequence similarity estimation via lexicographic comparison of hashes

**DOI:** 10.1093/bioinformatics/btad652

**Published:** 2023-10-25

**Authors:** Grant Greenberg, Aditya Narayan Ravi, Ilan Shomorony

**Affiliations:** Department of Electrical and Computer Engineering, University of Illinois at Urbana-Champaign, Urbana, IL, United States; Department of Electrical and Computer Engineering, University of Illinois at Urbana-Champaign, Urbana, IL, United States; Department of Electrical and Computer Engineering, University of Illinois at Urbana-Champaign, Urbana, IL, United States

## Abstract

**Motivation:**

Pairwise sequence alignment is a heavy computational burden, particularly in the context of third-generation sequencing technologies. This issue is commonly addressed by approximately estimating sequence similarities using a hash-based method such as MinHash. In MinHash, all *k*-mers in a read are hashed and the minimum hash value, the min-hash, is stored. Pairwise similarities can then be estimated by counting the number of min-hash matches between a pair of reads, across many distinct hash functions. The choice of the parameter *k* controls an important tradeoff in the task of identifying alignments: larger *k*-values give greater confidence in the identification of alignments (high precision) but can lead to many missing alignments (low recall), particularly in the presence of significant noise.

**Results:**

In this work, we introduce LexicHash, a new similarity estimation method that is effectively independent of the choice of *k* and attains the high precision of large-*k* and the high sensitivity of small-*k* MinHash. LexicHash is a variant of MinHash with a carefully designed hash function. When estimating the similarity between two reads, instead of simply checking whether min-hashes match (as in standard MinHash), one checks how “lexicographically similar” the LexicHash min-hashes are. In our experiments on 40 PacBio datasets, the area under the precision–recall curves obtained by LexicHash had an average improvement of 20.9% over MinHash. Additionally, the LexicHash framework lends itself naturally to an efficient search of the largest alignments, yielding an O(n) time algorithm, and circumventing the seemingly fundamental $O(n^{2})$ scaling associated with pairwise similarity search.

**Availability and implementation:**

LexicHash is available on GitHub at https://github.com/gcgreenberg/LexicHash.

## 1 Introduction

Sequence alignment is an important first step in the analysis of sequencing data, particularly in the context of third-generation sequencing platforms, which produce long reads, but with high error rates. The presence of errors in the reads (substitutions, insertions, and deletions) makes the task of identifying regions of similarity between pairs of reads computationally intensive. Given a sequencing dataset with *n* reads of length ℓ, performing standard dynamic-programming-based alignment algorithms [such as Smith–Waterman (Smith and Waterman 1981)] for each pair of reads requires $O(n^{2}\ell^{2})$ time, which is intractable when *n* and ℓ are large.

One way to alleviate this problem is to note that, in many applications, one is only interested in identifying pairs of reads with a “significant” overlap or a “large” alignment score. Moreover, the vast majority of read pairs typically do not have any overlap, and estimating their alignment score precisely is unnecessary. Based on these insights, the pairwise alignment problem is solved in practice using a two-step approach: first, hashing-based methods are used to coarsely estimate pairwise similarities scores in a computationally efficient way; then, more precise alignment algorithms are applied only to pairs that are identified as likely to have a significant alignment (Chaisson and Tesler 2012, Berlin *et al.* 2015, Ondov *et al.* 2016, Jain *et al.*, 2018).

A popular approach for the first step, similarity estimation, is to estimate the fraction of *k*-mers shared by each pair of reads (i.e. the *k*-mer Jaccard similarity). In practice, this can be efficiently performed using a form of locality-sensitive hashing called MinHash. In the MinHash paradigm (Broder 1997), we apply a hash function to each *k*-mer in a read and take only the minimum value across all the computed hashes (i.e. the min-hash). This allows us to generate a *sketch* for each read, consisting of the min-hashes computed for several different hash functions (Ondov *et al.* 2016, Shaw and Yu 2021). Given the sketches of two reads, one can estimate how similar the reads are by counting the fraction of matching min-hashes between the sketches. This can be shown to provide an unbiased estimate of the Jaccard similarity between the reads (assuming hash functions are randomly generated). With *m* hash functions, the sketch of all reads can be computed in time O(nmℓ), and the pairwise similarity estimates can be computed in $O(n^{2}m)$, which is significantly faster than $O(n^{2}\ell^{2})$ for moderate values of *m*. This leads to significant computational savings in practice (Broder 1997, Li 2016, Jain *et al.* 2018).

**Figure 1.**
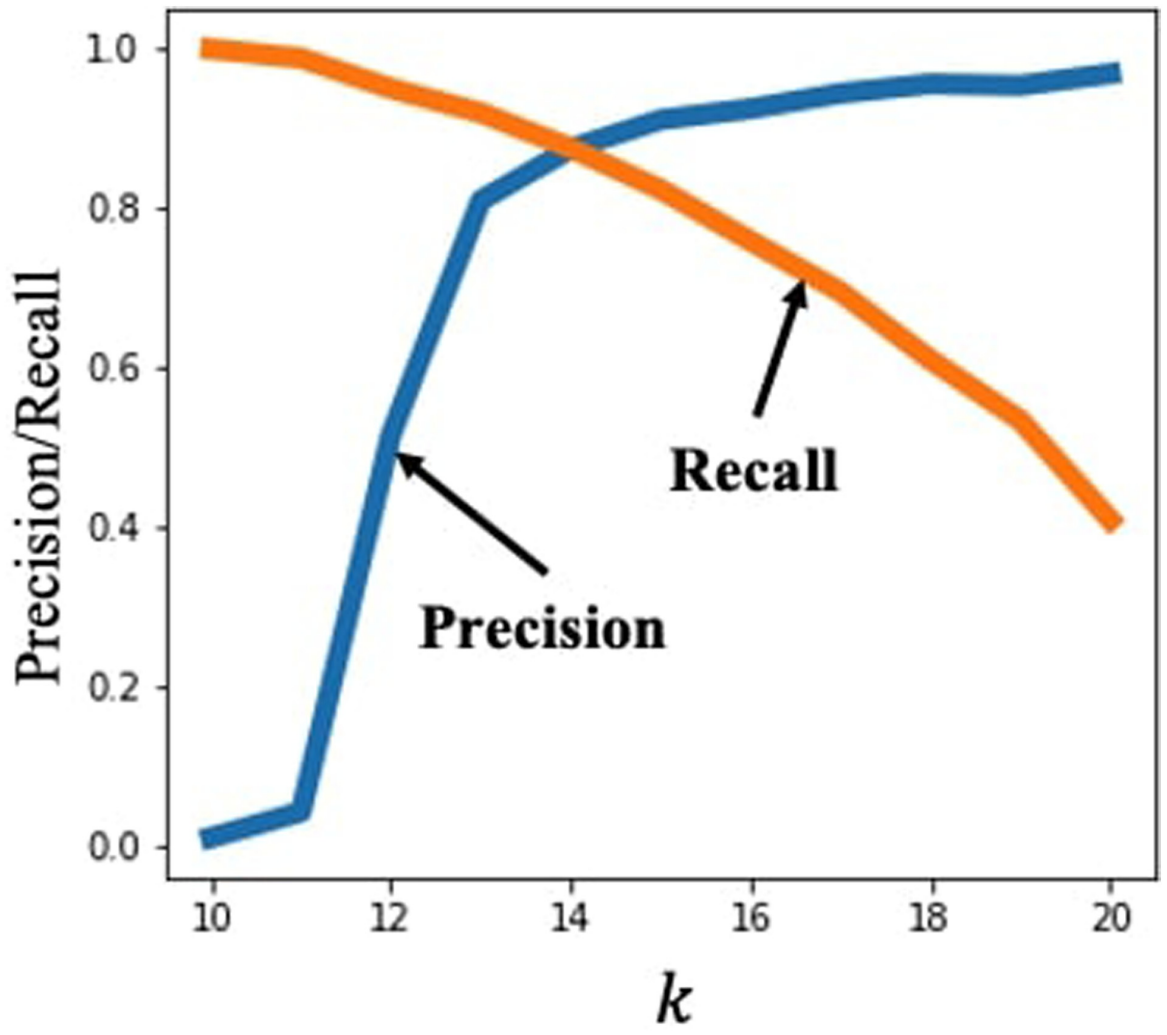
MinHash was used with 500 hash functions to find read overlaps for the NCTC4133 (*Staphylococcus epidermidis* genome) read dataset (Public Health England, Pacific Biosciences, and Wellcome Sanger Institute 2014), varying the value of *k*. The precision–recall tradeoff is apparent as precision is poor for smaller *k*, and recall is poor for large *k*. The alignment score threshold was set to 300.

The choice of the parameter *k* significantly affects the overall performance of MinHash by controlling an important tradeoff. When *k* is large, a matching min-hash between two reads is more meaningful, as it implies the presence of a longer matching substring. However, depending on the read error rates, picking a large *k* may lead to most *k*-mers containing an error, reducing the probability of a min-hash match between two reads with a true overlap. As such, a larger value of *k* leads to a higher *precision*, as we have more confidence in the identified pairs, but lower *recall*, as more true alignments may be missed. This is illustrated in Fig. 1.

The choice of *k* depends on the dataset and specific requirements for precision or recall. A higher error rate in the sequencing platform may require us to choose lower *k* values, to make sure that a reasonable fraction of *k*-mers is not corrupted by errors. On the other hand, if the composition of *k*-mers in the genome being sequenced is highly skewed, e.g. due to a significant nucleotide bias [as in the case of *Plasmodium falciparum*, where the A, T fraction is 80.6% (Vembar *et al.* 2016)], it may be preferable to choose a larger value of *k*. This is because non-overlapping reads are more likely to share common *k*-mers, and a matching min-hash is thus less meaningful (Baharav *et al.* 2020). Given the tradeoff controlled by the choice of *k*, a natural question is whether it is possible to modify MinHash to be less sensitive to the choice of *k*.

In this work, we introduce LexicHash, a new approach to pairwise sequence similarity estimation that combines the sketching strategy of MinHash with a lexicographic-based hashing scheme. While LexicHash still requires the choice of a parameter $k_{\text{max}}$, this value just represents an upper bound on *k*, and the performance is nearly unaffected by its value. This is because LexicHash identifies variable-length substring matches (for lengths below $k_{\text{max}}$) between the reads, simultaneously achieving the high precision of large *k* and the high recall of small *k*.

The LexicHash algorithm first generates a set of “masks,” which are length-$k_{\text{max}}$ strings of symbols from {A, C, G, T}. Each mask determines a different lexicographic ordering on $k_{\text{max}}$-mers. For a given mask, the hash-value of a $k_{\text{max}}$-mer is its lexicographic ranking according to the mask. Analogous to MinHash, a sketch of a read is created by storing the min-hashes for different mask choices. However, the similarity between two reads is not the number of min-hash exact matches across the sketches, as in MinHash. Instead, given the LexicHash sketches of two reads, it is possible to efficiently compute the length of the prefix match of the corresponding $k_{\text{max}}$-mers. This way, LexicHash can identify variable-length substring matches between reads from their sketches. The sketches are also constructed in such a way that, to compare sketches, we can traverse the sketches position-by-position, as with the MinHash sketch.

In addition to the sketching procedure, we present an efficient method to compute the maximum match length simultaneously for all pairs of reads by building prefix trees on the set of lexicographic first $k_{\text{max}}$-mers. In terms of the number of sequences *n*, the prefix tree method can find the top *T* similar read pairs, in O(n) time, for T=O(n), circumventing the $O(n^{2})$ time complexity inherent to the vanilla MinHash scheme.

We apply the LexicHash approach to read data from 40 genomes from the NCTC collection of Public Health England (Public Health England, Pacific Biosciences, and Wellcome Sanger Institute 2014), as well as read data from the *P.falciparum* (Vembar *et al.* 2016) and *Escherichia coli* genomes. We use minimap2 (Li 2018) and Daligner (Myers 2014) to create a ground truth of alignment sizes for all pairs of reads. Our results indicate a significant improvement over the standard MinHash approach. We further provide a brief comparison to minimizers and the highly optimized strobemers method for sequence similarity detection (Sahlin 2021a).

### 1.1 Related work

MinHash was introduced by Broder (1997), Broder (1997) as a method to probabilistically estimate the Jaccard similarity between documents. Starting with MHAP (Berlin *et al.* 2015), the MinHash paradigm has been successfully applied to the problem of sequence alignment of genomic sequencing data in several settings (Ondov *et al.* 2016, Popic and Batzoglou 2016, Koren *et al.* 2017, Jain *et al.* 2018). The related idea of minimizer schemes was introduced by Schliemer *et al.* (Schleimer *et al.* 2003) for document similarity, and by Roberts *et al.* (2004) for biological sequence alignment. Minimizer methods use representative *k*-mers, called minimizers, as seeds for alignment. Minimizers are chosen based on an ordering of the set of all *k*-mers. In this way, minimizer methods are similar to MinHash, but in addition, they incorporate a window guarantee, where consecutive minimizers are no farther apart than a window size *w*, which helps in the accuracy of the “chaining” step, in which chains of minimizers are identified (Li 2016, 2018).

Important variations of the minimizers approach as well as alternate methods have been proposed, including *strobemers* (Sahlin 2021a,b, Maier and Sahlin 2023), which link minimizers of short *k*-mers and showed more robustness to distinct mutation rates and indels (additionally, minimizers can be applied on top of the strobemer sampling); FracMinHash (Brown and Irber 2016, Irber *et al.* 2022), which creates sequence sketches of the smallest fraction of hash-values to estimate containment between sequences; *syncmers* (Edgar 2021, Dutta *et al.* 2022), which find minimizers of substrings within *k*-mers; *weighted k*-mers (Jain *et al.* 2020), which assigns higher weights to infrequent *k*-mers; and *fuzzy* seed matching (Firtina *et al.* 2023), which can match seeds in the presence of sequencing errors. Some methods consider the problem of selecting minimizers specifically with the aim of reducing the *density* (i.e. fraction of *k*-mers selected) and increasing conservation (Orenstein *et al.* 2017, Marçais *et al.* 2018, DeBlasio *et al.* 2019, Zheng *et al.* 2020, Shaw and Yu 2021). Others directly target efficient genome assembly (Chin and Khalak 2019, Ekim *et al.* 2021). In addition, some works have studied ways to use minimizer-based schemes to estimate specific measures of sequence similarity such as edit distance (Marçais *et al.* 2019, Joudaki *et al.* 2020) and Spectral Jaccard Similarity (Baharav *et al.* 2020).

The idea of using masks to generate different lexicographic orderings and using those to sort suffixes was proposed in a recent work (Shomorony and Kamath 2021), which used this idea to study information-theoretic questions regarding the rate-distortion tradeoff in sequence alignment. The present work can be seen as building on these theoretical ideas to propose an actual similarity estimation algorithm that can be applied to read data sets in practice. As future work, we will evaluate the effectiveness of LexicHash in a minimizer scheme, for instance, to create sketches on the minimizer space itself as in MashMap (Jain *et al.* 2018).

## 2 Materials and methods

In order to put in perspective the construction of LexicHash sketches, we first briefly describe the standard MinHash framework. We then present the LexicHash sketching approach, and an efficient method of doing pairwise comparison.

For a parameter *k*, consider the set Γ(s) of all length-*k* substrings (*k*-mers) of a sequence *s*. The MinHash algorithm picks *m* distinct hash functions ${\left\{\phi_{i}\right\}}_{i=1}^{m}$, each of which maps a *k*-mer to an integer. For each hash function $\phi_{i}$, the minimum hash value (min-hash) $h_{i}$ is computed as


(1) $$h_{i}=\underset{x\in \Gamma (s)}{\text{arg}\text{min}}\phi_{i}(x).$$

By concatenating the min-hashes for i=1,…,m, we obtain a “sketch” $\text{Sk}(s)=[H_{\text{ovlp}},h_{2},\ldots ,h_{m}]$.

Intuitively, when two reads $s_{1}$ and $s_{2}$ have a significant overlap, it is likely that many *k*-mer entries in $\text{Sk}(s_{1})$ will match the corresponding entry in $\text{Sk}(s_{2})$. Thus, a reasonable measure of similarity between $s_{1}$ and $s_{2}$ can be computed by traversing the sketches position-by-position and counting the number of matching *k*-mers. It can be verified that the fraction of matching min-hashes provides an unbiased estimate of the *k*-mer Jaccard similarity (Berlin *et al.* 2015); that is,


$$E\left[\frac{1}{m}\sum_{i=1}^{m}1\left\{h_{i}^{\left(1\right)}=h_{i}^{\left(2\right)}\right\}\right]=J(s_{1},s_{2})\;:=\frac{\left|\Gamma (s_{1})\cap \Gamma (s_{2})\right|}{\left|\Gamma (s_{1})\cup \Gamma (s_{2})\right|}.$$

The key advantage of this procedure, as opposed to comparing entire sets of *k*-mers of two reads, is that we can compare the corresponding sketch entries one at a time, a much more efficient computation. Note that MinHash operates on fixed-length *k*-mers, which requires us to pick the value of *k* prior to utilizing this method, imposing the tradeoff described in Section 1.

### 2.1 LexicHash

LexicHash is similar to MinHash in that distinct hash functions are used to create sketches of a sequence by storing the vector of minimum hash values over all *k*-mers in the sequence. However, the *k*-value used in LexicHash actually corresponds to a *maximum* match length $k_{\text{max}}$, and the hashing scheme maintains the ability to capture any match-length below the chosen $k_{\text{max}}$.

**Figure 2.**
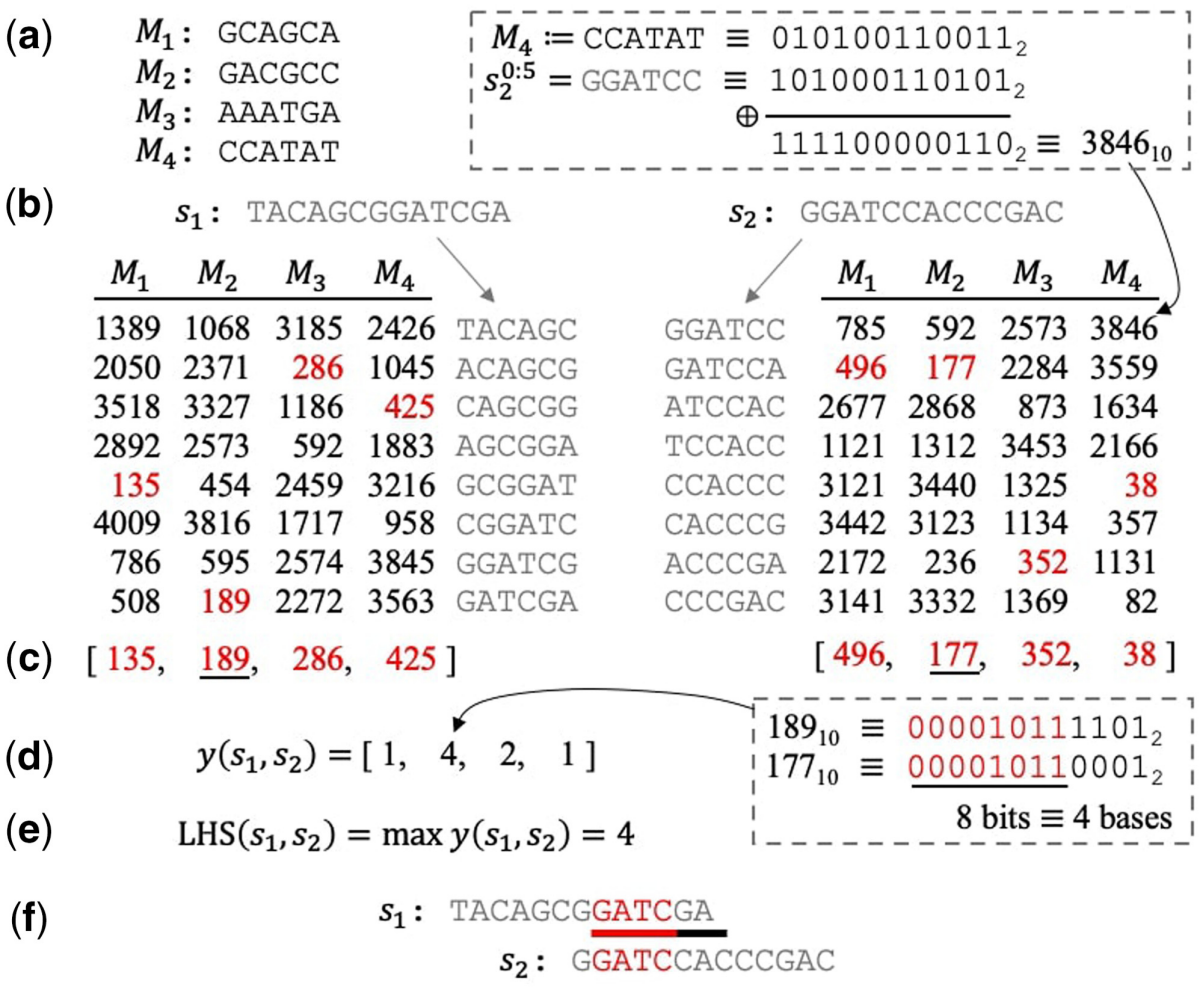
LexicHash pipeline. (a) Four masks are randomly generated for $k_{\text{max}}=6$. All mask and read bases, A, T, C, G, are mapped to bits 00,01,10,11, respectively. (b) Hash-values are computed for all constituent $k_{\text{max}}$-mers of two length-13 reads across all masks. An example calculation is given for the fourth mask and first $k_{\text{max}}$-mer of $s_{2}$, where a simple XOR operation is applied to the binary representations of $M_{4}$ and $s_{2}^{0:5}$, resulting in the lexicographic ranking (3846st) according to the mask. (c) The sketches of $s_{1}$ and $s_{2}$ consist of their minimum lexicographic rankings (min-hashes) across all masks. (d) The vector of match lengths between the reads, $y(s_{1},s_{2})$, is determined by counting the matching bits in the prefixes of each pair of min-hashes in the sketches. For example, the similarity score (i.e. match-length) corresponding to the second mask is four, since the binary representations of the min-hashes, 189 and 177, share eight bits. (e) The similarity score of 4 is the maximum of all match-lengths. (f) The similarity score represents a true 4-mer match between the two sequences. To illustrate the effectiveness of LexicHash, a sequencing error was inserted in the length-7 overlap between $s_{1}$ and $s_{2}$; the lexicographic-based min-hashes allow for the detection of the matching 4-mer, but the MinHash scheme for k=6 would not capture any similarity.

The LexicHash scheme utilizes *m masks*, each of which is a length-$k_{\text{max}}$ sequence over {A, C, G, T}, as shown in Fig. 2a where m=4 and $k_{\text{max}}=6$. To compute hash-values, all bases in the masks and sequences, A, C, G, and T, are mapped to bits 00, 01, 10, and 11, respectively. For a mask *M*, the hash-value of a $k_{\text{max}}$-mer *x* is simply the bitwise XOR between *M* and *x*, which can be seen as a $2k_{\text{max}}$-bit integer. Notice that, for the all-zeros mask, the hash integer value can be thought of as the $k_{\text{max}}$-mer’s lexicographic rank (among all $k_{\text{max}}$-mers). For other masks, the hash value can be similarly thought of as a ranking that uses a different lexicographic ordering for each position of the string. An example is given in the top-right of Fig. 2, where the hash-value of the first 6-mer of $s_{2}$ is computed according to the fourth mask, $M_{4}$. For each mask $M_{i}$, the minimum lexicographic rank of a sequence *s* is computed as


(2) $$r_{i}=\underset{x\in \Gamma (s)}{\text{arg}\text{min}}M_{i}⊕x,$$

splitting ties arbitrarily. The sketch of *s* is then $\text{Sk}(s)=[r_{1},r_{2},\ldots ,r_{m}]$, as shown in Fig. 2c. The time complexity of the sketching procedure is the same as that of MinHash, O(nmℓ), where ℓ is the average sequence length. Despite operating on longer *k*-mers, sketching is potentially more computationally efficient than typical MinHash sketching due to the speed of the bitwise XOR operation.

To estimate the similarity between a pair of sequences, we traverse the two sketches position-by-position and determine the match lengths. For a pair of min-hashes at index *i* in the sketch, $r_{i}^{\left(1\right)}$ and $r_{i}^{\left(2\right)}$, the match length is simply the length of the longest matching prefix of their binary representations. An example is shown in Fig. 2d, where $r_{i}^{\left(1\right)}=189$ and $r_{i}^{\left(2\right)}=177$, and the match length is four bases since the first eight bits of the ranks match. The real corresponding match is shown in red in Fig. 2f. After performing this process on each of the *m* pairs of min-hashes we obtain a vector of match-lengths $y(s_{1},s_{2})$, as seen in Fig. 2d. Note that in the MinHash scheme, the similarity score is computed in an “all or nothing” manner, where a pair of min-hashes only count if they match exactly. In LexicHash, pairs of min-hashes correspond to match lengths that can contribute to the overall similarity score regardless of equality. This makes the choice of $k_{\text{max}}$ nearly irrelevant for the resulting vector $y(s_{1},s_{2})$.

**Figure 3.**
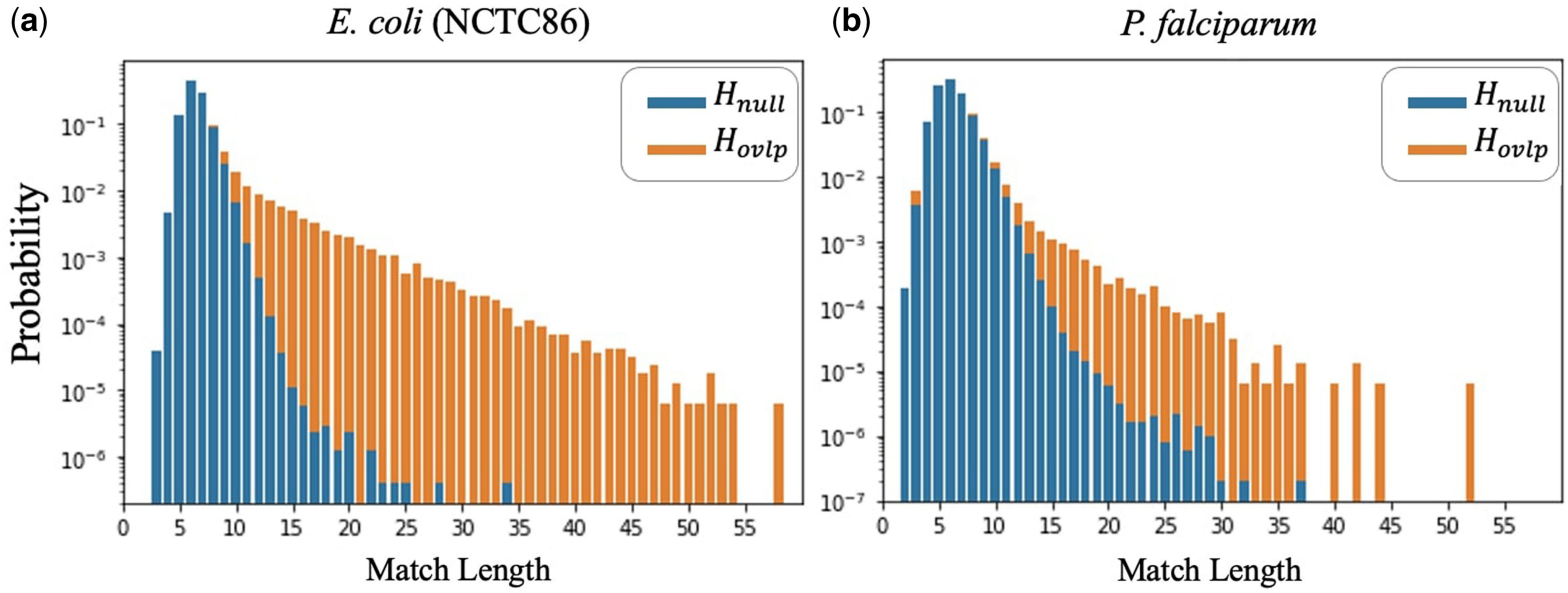
Empirical PMFs of match lengths for $H_{\text{null}}$ (non-overlapping reads) versus $H_{\text{ovlp}}$ (overlapping reads with alignment fraction >0.2). The PMF in (b) has a heavier tail for $H_{\text{null}}$ and lighter tail for $H_{\text{ovlp}}$, indicating a more challenging dataset than that of (a). The genome of (b), *P.falciparum*, has a very low *GC*-content, which increases the match lengths for non-overlapping reads.

Intuitively, the vector of match lengths $y(s_{1},s_{2})$ can be thought as the sequence of observations for a binary hypothesis test between hypotheses “$H_{\text{null}}$: there is no significant alignment between $s_{1}$ and $s_{2}$” and “$H_{\text{ovlp}}$: there is a significant alignment between $s_{1}$ and $s_{2}$.” To visualize this hypothesis test problem, in Fig. 3, we plot the probability mass functions (PMFs) of the match lengths under the two hypotheses for two different datasets. Notice that the match length distributions under $H_{\text{null}}$ and $H_{\text{ovlp}}$ have significantly different tails, with the tail under $H_{\text{ovlp}}$ being significantly heavier. This suggests that a good test statistic for $H_{\text{null}}$ versus $H_{\text{ovlp}}$ is simply the maximum value of the vector $y(s_{1},s_{2})$. Hence, we define the LexicHash score as


(3) $$\text{LHS}(s_{1},s_{2})=\max\;y(s_{1},s_{2}),$$

which provides as a measure of similarity between $s_{1}$ and $s_{2}$. While this test statistic may not be theoretically optimal—if the PMFs were known, an optimal decision rule would be the likelihood ratio test—it suffices to obtain a significant improvement in performance over MinHash, and allows for the runtime improvements described in Section 2.2.

We point out that the tail of the match length distribution under $H_{\text{null}}$ is different for different datasets, as shown in Fig. 3. In particular, how heavy the tail is under $H_{\text{null}}$ can be thought of as a measure of the difficulty of identifying alignments in a given dataset. For example, the genome in Fig. 3b, *P.falciparum*, the malaria parasite, is a notoriously difficult genome to assemble (Vembar *et al.* 2016). At a high level, the reason is that the GC content in the genome is very low, meaning that *k*-mers with high AT content are fairly common, which leads to many substring matches between non-overlapping reads. As such, $H_{\text{null}}$ in Fig. 3b has a heavier tail than in Fig. 3a.

### 2.2 Efficient pairwise read comparison via prefix trees

Given the sketches $\text{Sk}(s_{i})$ for all reads $s_{1},\ldots ,s_{n}$, one still needs to perform $\left(\begin{array}{c} n\\ 2 \end{array}\right)$ pairwise comparisons to compute $\text{LHS}(s_{i},s_{j})$ for each pair. For the downsampled datasets used in the results in Section 3 with $n∼10^{3}$, this can still be done quickly. For large datasets, however, the quadratic scaling with *n* makes this step slow. In this section, we show how the LexicHash framework is amenable to an efficient search for pairs $\left(s_{i},s_{j}\right)$ with large values of $\text{LHS}(s_{i},s_{j})$. In particular, we describe an algorithm that finds the top-*T* values of $\text{LHS}(s_{i},s_{j})$ and avoids the $O(n^{2})$ scaling when T=O(n).

**Figure 4.**
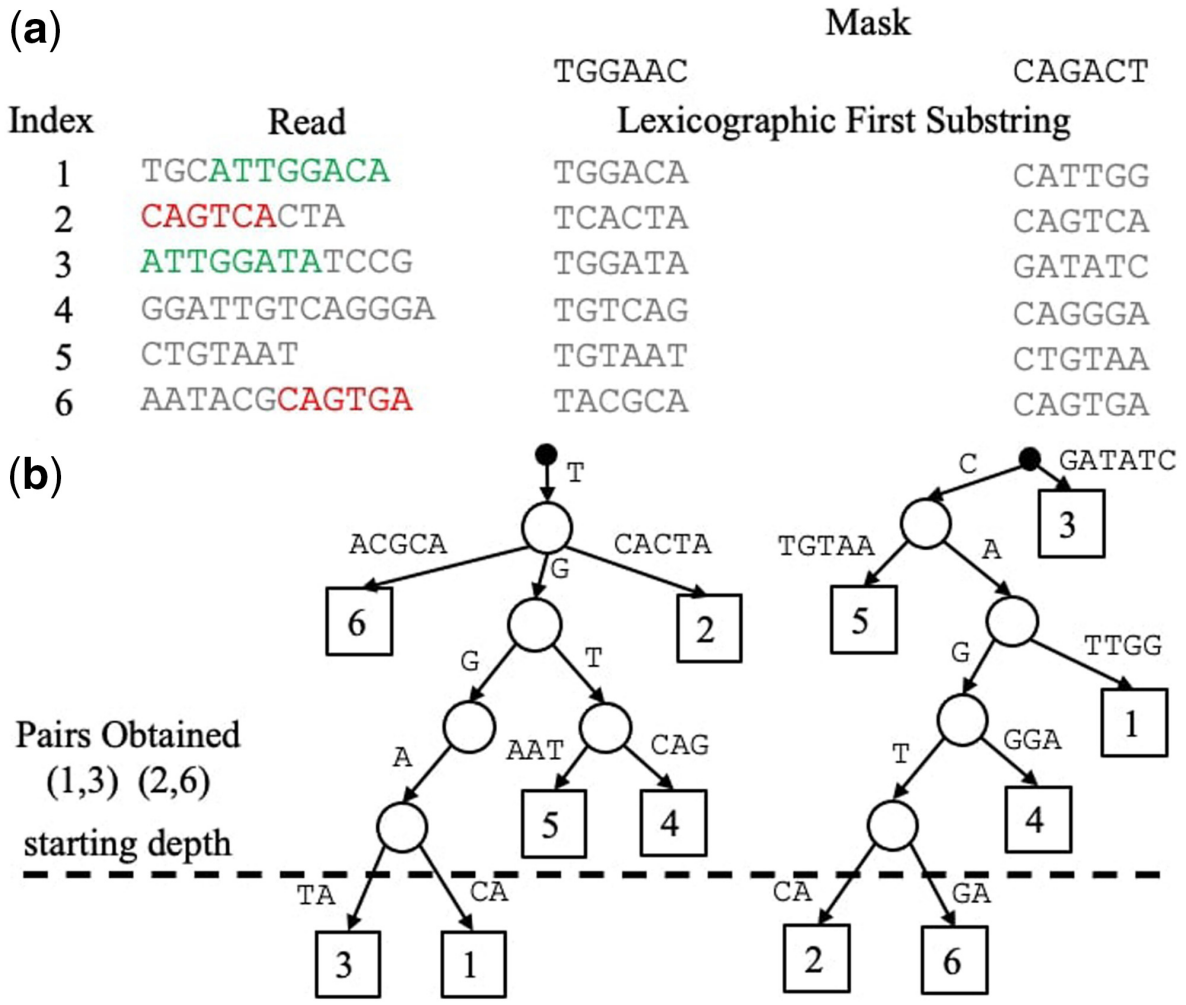
(a) Table of lexicographic first substrings (of length $k_{\text{max}}=6$) for six reads across two lexicographic orders, specified by a mask (explained in Fig. 2). Two significant read overlaps are indicated in read and green, and include sequencing errors. (b) Prefix Trees obtained from the lexicographic first $k_{\text{max}}$-mers for each mask. We use bases here for clarity, but our implementation uses binary representations. To collect pairs of reads with potential overlaps, we start at the maximum depth, four in this case, and add each pair of reads below. Note how the use of different masks enables the discovery of all significant overlaps.

Suppose one is interested in finding the top-*T* pairs of most similar sequences, i.e. the *T* largest values of $\text{LHS}(s_{i},s_{j})$ (e.g. we could set T=10n, to find roughly 10 alignments per read). For each mask $M_{i}$, we build a prefix tree data structure, as shown in Fig. 4. Each prefix tree is built using the min-hash values of all *n* sequences in binary form. Each level of a tree requires a partition of all subtrees, which consists of at most *n* elements. Thus, the time complexity of building all prefix trees is at most $O(nmk_{\text{max}})$.

At a depth of *h* in the prefix tree, each subtree contains $k_{\text{max}}$-mers whose length-*h* prefixes match exactly, indicating a likely overlap for large *h*. Hence, we start at a depth of $h=k_{\text{max}}$, collect all pairs of substrings in each subtree at that depth (simultaneously across all prefix trees). Then we iteratively decrement *h* and continue collecting pairs (which have not already been added) until the target number of pairs, *T*, have been collected. Note that any single pair may only be seen a maximum of $mk_{\text{max}}$ times, i.e. once at each depth for each tree. Thus, even in the worst case, the overall time complexity for building and aggregating pairs is $O(nmk_{\text{max}}+Tmk_{\text{max}})$. Importantly, when T=O(n), this avoids the natural $O(n^{2})$ scaling associated with having to find significant pairs among a total of $\left(\begin{array}{c} n\\ 2 \end{array}\right)=O(n^{2})$ pairs.

We point out that, in the standard MinHash paradigm, one could also try to mitigate the burden of having to compare all pairs of sketches. This can be done by first creating a list of all computed min-hash values for each hash function. Then, a match count table for read pairs can be computed by going through the list of min-hash values and, for each one, incrementing the count of each pair of reads that share that min-hash value. However, this approach still has a $O(n^{2})$ worst-case time complexity since, in principle, there can be a set of $\Theta (n^{2})$ pairs each of which shares at least one min-hash (which would lead to $\Theta (n^{2})$ increment operations).

The memory requirement of the prefix tree method is relatively low at $O(nmk_{\text{max}})$. The algorithm used in the current implementation is presented in Supplementary Algorith C1. It takes advantage of the fact that the full prefix tree structure need not be stored, only a partition of sequence indices at each level of the tree. Furthermore, a $k_{\text{min}}$ can be set so that only partitions at depths $h\in [k_{\text{min}},k_{\text{max}}]$ are stored. Lastly, singleton subtrees also do not need to be stored. These improvements greatly reduce the memory requirements of LexicHash.

## 3 Results

We present the results in three subsections. The first compares the similarity estimation performance of LexicHash and MinHash in terms of the receiver operator characteristic (ROC) and precision–recall curves (PRC). The second evaluates the runtime performance of LexicHash, validating the prefix-tree method described in Section 2.2. Finally, in Section 3.3, we provide a brief performance comparison to a basic minimizer method as well as the state-of-the-art strobemers seeding method.

### 3.1 Performance comparison of LexicHash and MinHash

To compare LexicHash and MinHash, we perform read similarity estimation on 41 PacBio datasets, 40 from NCTC (Public Health England, Pacific Biosciences, and Wellcome Sanger Institute 2014), and also a *P. falciparum* dataset. The datasets have a wide range of nucleotide compositions. In particular, *P. falciparum*, the malaria parasite, has a very low GC content, leading to long stretches of As and Ts. Each dataset is downsampled to $n∼10^{3}$ reads, such that each read overlaps with roughly 0.5–2% of all reads after preprocessing. More details on the preprocessing steps used to create the datasets are explained in Supplementary Section B. We generate a ground truth for each dataset using DALIGNER (Myers 2014) for the NCTC datasets and minimap2 (Li 2018) for the *P.falciparum* dataset. Each ground truth consists of a set of overlapping reads, using a threshold of 0.2 for the alignment fraction (defined as $\frac{a}{\ell_{1}+\ell_{2}-a}$ for reads with lengths $\ell_{1}$ and $\ell_{2}$ overlap size *a*).

We use a vanilla version of MinHash, without any algorithmic or heuristic improvements (such as those used in MHAP and Minimap2), to obtain a fair conceptual comparison of the two hash-based sketching approaches. We run MinHash on several appropriate values of *k*, ranging from 7 to 16 (MHAP default). A maximum match length of $k_{\text{max}}=32$ is used by default for LexicHash. In a more memory-efficient implementation of LexicHash (e.g. in C++), using 2-bit encoding for the masks, this could leverage 64-bit architectures, but our current implementation does not take advantage of that. In such an efficient implementation, a $k_{\text{max}}>32$ would increase runtime and memory usage of LexicHash due to the need for more than one integer per hash-value. In the context of pairwise long-read similarity estimation, our results indicate that $k_{\text{max}}>32$ does not improve the accuracy. For highly accurate reads, $k_{\text{max}}>32$ may be necessary, but would not limit the functionality of LexicHash beyond the increase in runtime (see Supplementary Fig. S8). Masks are generated uniformly at random over {A, C, G, T}. In Section 4, we discuss more sophisticated possibilities for mask generation. To account for reverse complement matches, we include for each dataset D, the set of reverse complement reads $\bar{D}$. Sketches are created for all reads in $D\cup \bar{D}$; sequence similarity estimation is then performed for read pairs within the original dataset D↔D, and pairs of one original read and one complemented $D↔\bar{D}$.

**Figure 5.**
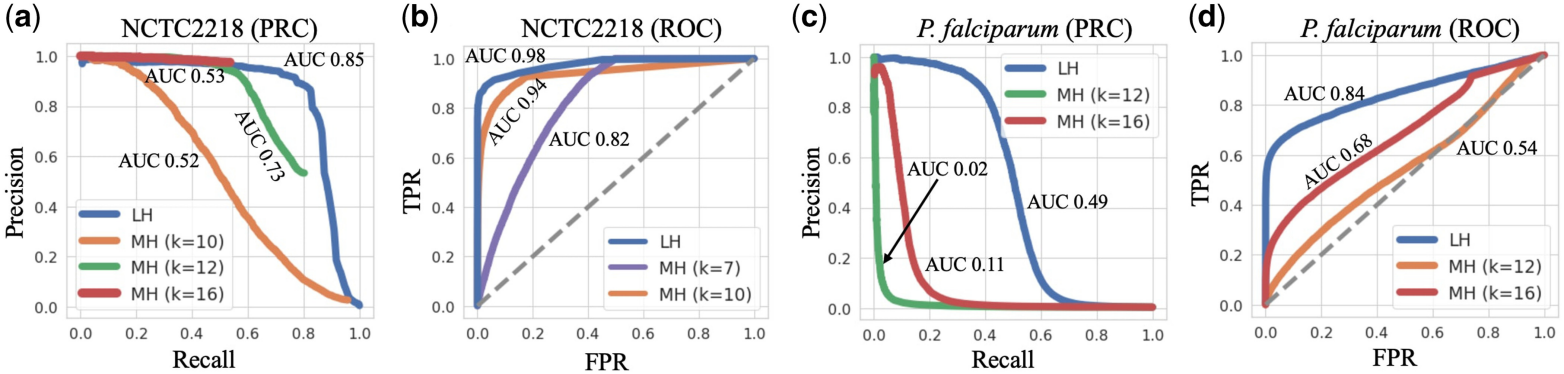
ROC and PRC plotted for the NCTC2218 (*S.pyogenes* genome) dataset (a, b) and the *P.falciparum* dataset (c, d). The blue lines represent the performance of LexicHash (LH), and the purple, orange, green and red, those of MinHash (MH) for k=7,10,12,16, respectively. In all plots, LexicHash outperforms MinHash as measured by area under the curve (AUC). In the case of the challenging *P.falciparum* dataset, MinHash performs close to random guessing (dashed line in (c) where TPR=FPR), and has almost no signal in the PRC of (d).

**Figure 6.**
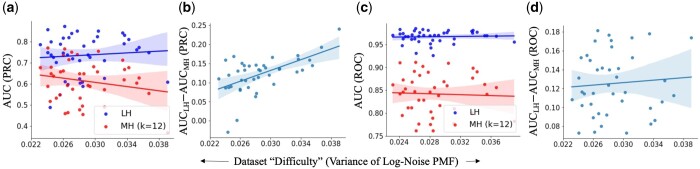
AUC comparison between LexicHash and MinHash across 40 NCTC datasets (each point corresponds to one dataset). (a) The AUC of the ROC versus dataset “difficulty” as measured by the variance of the log of the noise (match lengths of non-overlapping read pairs) PMF. (b) Improvement in AUC (ROC) versus difficulty. The gap in performance is seen to increase somewhat linearly as the difficulty increases. (c, d) Same as (a, b) but for the PRC. The performance gap increases slightly with more difficulty.

For each dataset, we run LexicHash and MinHash with 100 hash functions, respectively. Overall for the 40 NCTC datasets, the area under the curve (AUC) of PRC and ROC for LexicHash increased by 20.9% and 14.7% on average, respectively, when compared to MinHash using k=12. Performance for a larger number of hash functions follows a similar pattern as that of Figs 5 and 6, but the performance gap from LexicHash to MinHash narrows (e.g. at 1000 hash functions, AUC-ROCs are all nearly 1 for the NCTC datasets). See Supplementary Section A for more results. In Fig. 5, we plot ROC curves and PRCs for two of the datasets, from the *Streptococcus pyogenes* (NCTC2218) and *P.falciparum* genomes. To be as conservative as possible, we identify and plot the *k*-value that achieves the greatest AUC for MinHash (k=12 for (a), k=10 for (b), and k=16 for (c, d)), as well as other *k*-value(s) for comparison. We note that, since the fraction of read pairs that have an overlap is small, the false positive rates tend to be low, but precision and recall are not directly affected.

Observing the results on the *S. pyogenes* dataset (NCTC2218) shown in Fig 5a and b, LexicHash (LH) performs better than MinHash (MH) consistently for different false positive rates, and overall with AUCs of 0.85 and 0.73 (k=12) for the PRC, and 0.98 and 0.94 (k=10) for the ROC. As expected, the large-*k* MinHash (k=16) achieves high precision, but is unable to find more than 55% of overlaps, which is why the PRCs seems to stop suddenly (see Davis and Goadrich, 2006 for more details on PRC behavior). On the other hand, the precision for small-*k* (k=10) degrades rapidly as the similarity threshold increases since small *k* captures spurious matches in non-overlapping read pairs. MinHash for k=12 strikes a balance, but its performance is inferior compared to LexicHash. Similarly, LexicHash greatly outperforms MinHash on the *P.falciparum* dataset, as shown in Fig 5c and d, with an AUC-PRC of 0.49 for LexicHash and only 0.11 for k=16 MinHash. It is worth noting that due to very high AT content, performing sequence alignment on the *P.falciparum* dataset is particularly difficult (Vembar *et al.* 2016), and the minimap2 “ground truth” may be far from perfect, which may partially explain the poor performance of both MinHash and LexicHash. Nevertheless, the improvement of LexicHash over MinHash is still quite meaningful.

As seen in Fig. 3, it becomes harder to differentiate between real overlaps (signal) and spurious ones (noise), when the noise PMF has a heavy right tail. In Fig. 6, we plot the AUC of ROC and PRC for all 40 NCTC datasets, as the size of the right tail increases. The measure of tail heaviness is captured by the Variance of Log of PMF under $H_{\text{null}}$ (The log was taken to amplify the variation in the *x*-axis. A similar, but less linear plot results in taking the regular variance of the noise PMF.), which is calculated based on histograms obtained from each dataset, such as those in Fig. 3. In particular, we notice that the MinHash AUC-PRC degrades in datasets with a heavier tail, while LexicHash performs consistently well. In datasets where the match length has a heavy right tail under $H_{\text{null}}$, differentiating between $H_{\text{null}}$ and $H_{\text{ovlp}}$ requires a method to detect long matches, and LexicHash is particularly effective at that.

### 3.2 Runtime comparison

As discussed in Section 1, the bottleneck typically lies in the pairwise comparison of sketches for larger datasets. Using a hash table for MinHash drastically reduces the required number of pairs for which to compute the similarity score. However, even assuming the number of reads sharing a hash value for a given hash function is a small percentage of the total number, the time complexity remains $O(n^{2}m)$. As an improvement over MinHash, we describe in Section 2.2 an O(nm) time algorithm to obtain the top-*T* most similar pairs of reads using LexicHash sketches. To compare the methods, we have both methods return the top T=5n.

For the runtime experiment, we perform similarity estimation on a full PacBio RS II *E. coli* dataset, which was used in a recent survey of long-read technologies (Tvedte *et al.* 2021). After filtering out reads with an average error rate of 30% or greater, there remain 113 378 reads. To evaluate the time complexity in practice, we create five new datasets of sizes 10, 30, 50, 70, and 90 thousand reads, by downsampling the full dataset. We compare runtimes using 100 hash functions each, k=12 for MinHash, and $k_{\text{max}}=32$ for LexicHash. Eighty CPUs are used to parallelize the sketching and pairwise comparison procedures for both methods.

**Figure 7.**
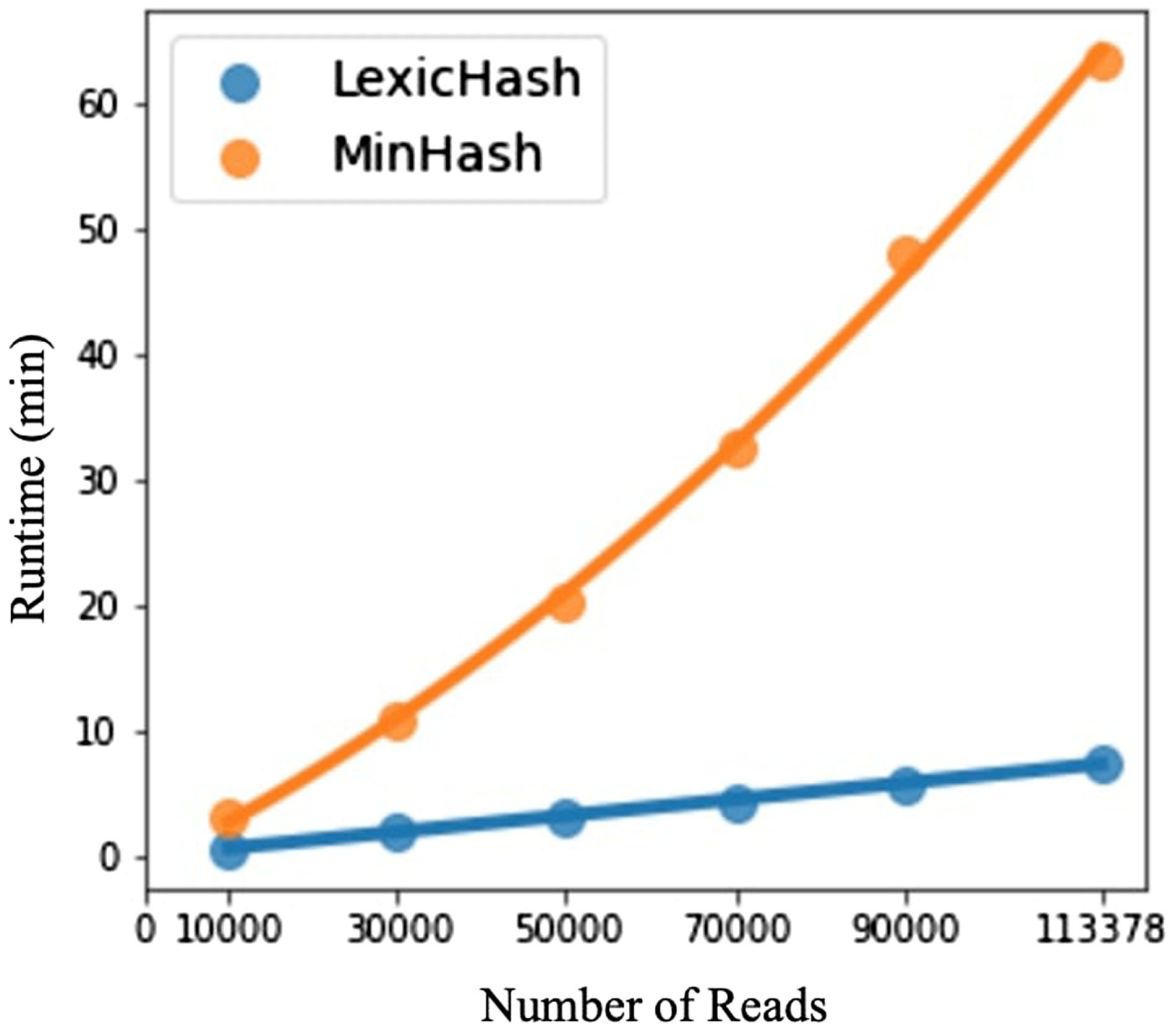
A comparison of runtimes between LexicHash and MinHash, with increasingly downsampled versions of an *E.coli* read dataset. For the task of finding the top-*T* most similar read pairs, the plots illustrate the O(n) time complexity of the LexicHash pipeline ($R^{2}=0.999$), compared to the inherent $O(n^{2})$ scaling for MinHash ($R^{2}=0.998$). To perform pairwise similarity estimation, the LexicHash method compares read sketches using the prefix tree method described in Section 2.2, which intrinsically “collects” the pairs with largest similarity scores first. Conversely, MinHash requires the calculation of all similarity scores before determining those with the top-*T* highest scores.

Shown in Fig. 7 are the total runtimes for both methods. LexicHash and MinHash appear to closely fit linear and quadratic curves, respectively, which is confirmed by the very high coefficients of determination ($R^{2}=0.999$ and 0.998). We stress that the absolute times for MinHash are not as important, as it is based on our Python implementation, which could be optimized. Instead, we argue that the experiment indicates that LexicHash avoids the $O(n^{2})$ time complexity of standard MinHash in the task of finding the most similar read pairs.

On the full *E.coli* dataset (2.4 GB of reads), LexicHash took 100.1 CPU-min, and 515.7 MB of RAM, when run on one CPU. MinHash took 339.3 CPU-min and 1.95 GB of RAM. For both methods, reads only need to be loaded one at a time in RAM to compute sketches. The memory consumption is favorable for LexicHash for this task because only 5n≈550,000 read-pairs need to be stored at maximum, whereas over 30 million read-pairs shared at least one *k*-mer for k=12 MinHash.

### 3.3 Comparison of LexicHash and minimizer methods

Although we view LexicHash as an improvement to the standard MinHash scheme and present performance comparisons with MinHash as a baseline, it is natural to try to compare the performance of LexicHash with other sequence similarity estimation methods. A fair comparison with MinHash is easily achieved by using the same number of hash functions in MinHash as in LexicHash; however, setting parameters for different methods in a fair manner is not as straightforward. Nevertheless, here we present a brief comparison between LexicHash and three methods: (i) the standard minimizer technique as presented by Roberts *et al.* (2004), (ii) the state-of-the-art strobemers method (Sahlin 2021a), and (iii) the FracMinHash method as implemented in the sourmash toolbox (Brown and Irber 2016, Irber *et al.* 2022).

Minimizer methods work by subsampling the *k*-mer set of a sequence so as to guarantee that at least one out of every *w* consecutive *k*-mers in the sequence will be sampled, for some choice of *k* and *w*. The sampled *k*-mers are called seeds. After the seeding step, in order to estimate the similarity between two sequences, a chaining step is performed with the goal of identifying chains of matching seeds with similar spacings across the different genomic sequences. For the standard minimizer scheme, we implement the chaining technique described in the minimap paper (Li 2016). See Supplementary Section B for more details. To obtain a crude similarity score, we use the number of “hits” (i.e. matching seeds) in the longest chain between two sequences. For strobemers, we use the StrobeMap (Sahlin 2021a) package using the *randstrobes* option. The similarity score is the sum of alignment sizes found between two sequences. For FracMinHash, given number of LexicHash hash functions *m*, we use an scale factor *s* which theoretically samples a similar number of *k*-mers from each read,


$$s=\frac{\bar{L}}{m},$$

where $\bar{L}$ is the average read length (typically 9–12 kpb). The corresponding estimated containment values are taken as the similarity measure itself.

After testing several parameter sets for all methods, we use those with the highest overall performance, which is (k,w)=(14,10) for minimizers, $\left(n,k,w_{\text{min}},w_{\text{max}})=(3,8,25,50\right)$ for StrobeMap, and k=16 for sourmash. Note that sourmash was not necessarily optimized for read-read similarity estimation.

**Figure 8.**
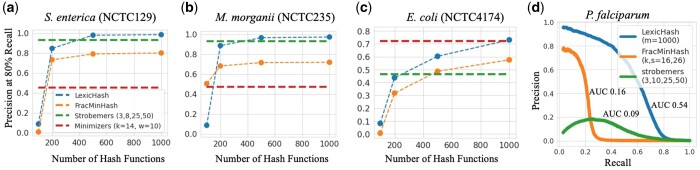
A comparison in performance between LexicHash, strobemers, FracMinHash, and the standard minimizer method. (a–c) The precision at 80% recall is plotted for increasing numbers of hash functions for LexicHash and FracMinHash (corresponding to a decreasing scale), and for strobemers and minimizers, represented by a dashed line, independent of the *x*-value. As the number of hash functions increases, the performance of LexicHash and usually FracMinHash becomes comparable to that of strobemers. (d) The precision–recall curve for the *P.falciparum* dataset is shown for LexicHash, strobemers, and FracMinHash. The similarity scores predicted by LexicHash is seen to better match the ground truth generated by minimap2.

In order to appropriately compare LexicHash with minimizer methods, it is useful to bear in mind the size of the sketch produced by each method (from which the sequence similarity is estimated). The number of *k*-mers sampled by minimizers is approximately 2ℓ/(w+1), where ℓ is the length of the sequence. For example, for a PacBio read of length 12 000 (the average read-length in the NCTC datasets), with w=10, the standard minimizer method would store nearly 2200 minimizer *k*-mers, and it would be arguably reasonable to compare it with LexicHash on as many masks, so that the sketch size is roughly of the same size. The strobemers sketch is of size nearly ℓ, since it samples at every position in the sequence. To show the comparison for different numbers of masks, in Fig. 8a–c, we provide a comparison of LexicHash to strobemers, FracMinHash, and standard minimizers as the number of LexicHash hash function increases. Furthermore, since in practice, one is more interested in the performance at larger recall values, we plot the precision at 80% recall [a number cited in the MHAP paper (Berlin *et al.* 2015)]. As the number of hash functions increases from 100 to 1000, the precision of LexicHash approaches that of strobemers. LexicHash appears to plateau in precision after a certain number of hash functions for certain datasets. For instance, in the NCTC129 and NCTC235 datasets, the precision is 0.99 and 0.98, respectively, at both 500 and 1000 hash functions. In most cases, the highly optimized strobemers method outperforms the standard minimizers method, but that is not always the case, as shown in Fig. 8c.

A particularly interesting comparison is on the challenging *P.falciparum* dataset. Most similarity estimation methods perform poorly on this dataset (and it is in fact unclear how good the ground truth provided by minimap2 is). However, by plotting the entire precision–recall curve, we see that LexicHash significantly outperforms strobemers and FracMinHash on recall values lower than 80%, and in general, with AUCs of 0.54, 0.09, and 0.16, respectively. This illustrates how LexicHash can be highly effective when the nucleotide composition is skewed.

## 4 Discussion

We present a new approach to sequence sketching and similarity estimation called LexicHash. As in the MinHash scheme, the method creates a sketch of a read based on min-hash values, for several hash functions based on distinct lexicographic orders. The LexicHash hashing scheme increases statistical power by effectively capturing matches of various sizes, thereby avoiding the precision–recall tradeoff of choosing a *k*-value in MinHash. Moreover, the lexicographic-based sketches of sequences utilized by LexicHash provide an efficient data structure to perform pairwise comparisons.

One idea to increase accuracy is to choose masks in a dataset-specific manner. For example, the *P.falciparum* genome has a greatly skewed nucleotide composition (specifically a very low GC content). Intuitively, this means that substrings of reads which have more Gs and Cs are more informative. Thus, a reasonable hypothesis is that if we choose lexicographic orders which favor G and C, the noise PMF might have a smaller tail, making it easier to distinguish from signal. Interestingly, however, we empirically show in Supplementary Section B that masks generated with a similar nucleotide composition as the dataset performs even better. We hypothesize that this is due to the GC content not being evenly distributed in real datasets (e.g. being concentrated in CpG islands). Alternatively, we could assign different weights to the masks, analogous to what is done for Spectral Jaccard Similarity (Baharav *et al.* 2020) or the weighted *k*-mer scheme (Jain *et al.* 2020).

The results in this article represent an initial exploration of the use of LexicHash as a sketching procedure for sequence similarity estimation. The results presented are conceptual in nature, as they focus on a comparison to a vanilla MinHash approach, and do not take into account a number of useful algorithmic techniques that can be used in combination with the scheme to improve the overall performance. A full exploration of LexicHash could also include other types of sequence alignment problems, including read-to-reference alignment and genome distance estimation such as Mash (Ondov *et al.* 2016), and applications outside of bioinformatics, such as plagiarism detection, where we search for similarities between text documents.

## Supplementary Material

btad652_Supplementary_Data

## Data Availability

The data underlying this article can be found at https://github.com/TavorB/spectral_jaccard_similarity (NCTC datasets), and in the Sequence Read Archive at https://www.ncbi.nlm.nih.gov/sra and can be accessed with accession numbers SRR3194822 *(P.**falciparum* dataset) and SRR3194822 *(E.**coli* dataset).
