# Supplementary material for: LexicHash: sequence similarity estimation via lexicographic comparison of hashes
Source: Bioinformatics. 2023 Oct 25;39(11):btad652. doi: 10.1093/bioinformatics/btad652 (PMC10628434; doi:10.1093/bioinformatics/btad652)
Supplement: btad652_Supplementary_Data [file btad652_supplementary_data.pdf]

Supplementary Information

A. Extended Results

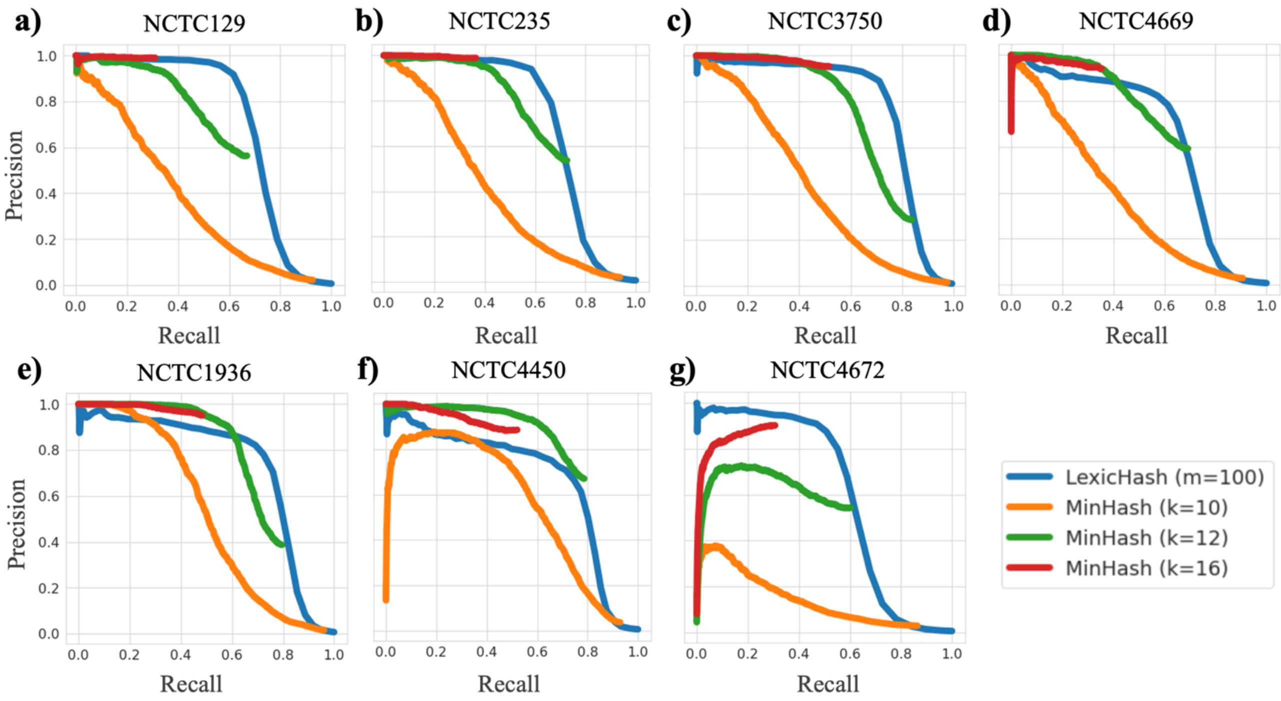

Figure S1. Precision-recall curves (PRC) illustrating four typical (a-d) and three interesting but rare phenomena (e-g). **(a-d)** NCTC129 (*Salmonella enterica*), NCTC235 (*Morganella morganii*), NCTC3750 (*Staphylococcus aureus*), NCTC4669 (*Streptococcus dysgalactiae*) datasets have typical PRC patterns, as seen in over 80% of NCTC datasets with which LexicHash was evaluated. **(e)** In the NCTC1936 (*Klebsiella pneumoniae*) dataset, LexicHash still outperforms MinHash in terms of AUC-PRC, but one can see that at lower recalls, the precision "droops" slightly. We hypothesize that LexicHash finds long matches (e.g., 40 bp) which may correspond to a short genomic repeat, and which minimap2 does not consider an overlap. **(f)** In the NCTC4672 (*Streptococcus uberis*) dataset, the performance of MinHash degrades rapidly at lower recalls. We hypothesize the cause is many inexact genomic repeats, in which LexicHash can find a long match, but which is too "noisy" for MinHash to find many matching k-mers. **(g)** In the NCTC4450 (*Escherichia coli*) dataset, higher thresholds on the alignment score lead to low precision, perhaps because of the overlap threshold of 0.2 used for the ground truth; LexicHash finds a long match, but often for read-pairs which "barely" overlap.

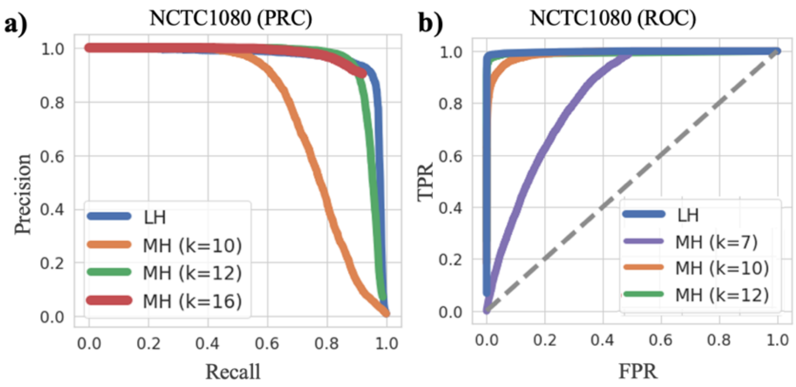

Figure S2. Typical precision-recall curve (PRC) and receiver operator characteristic (ROC) curve using 500 hash functions for an example NCTC dataset (*Streptococcus sp.*). Though LexicHash outperforms MinHash slightly, AUCs for both LexicHash and MinHash ($k = 12$) are quite high. More interesting and, we argue, more salient results are at 100 hash functions, since LexicHash is potentially both more computationally efficient and more accurate, and because LexicHash exhibits sufficient performance in a practical setting at a smaller number of hash functions.

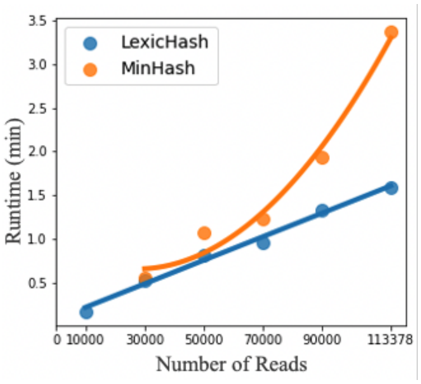

Figure S3. A comparison of runtimes for read-pair aggregation only (i.e., post-sketching) between LexicHash and MinHash (k=16). A linear fit for LexicHash has an $R^2 = 0.989$. A quadratic fit for MinHash has an $R^2 = 0.981$, which is a significantly better fit than a linear one, for which $R^2 = 0.908$. MinHash was unable to find $T = 5n$ read-pairs with a non-zero number of min-hash collisions for $n = 10{,}000$ reads, hence the missing data.

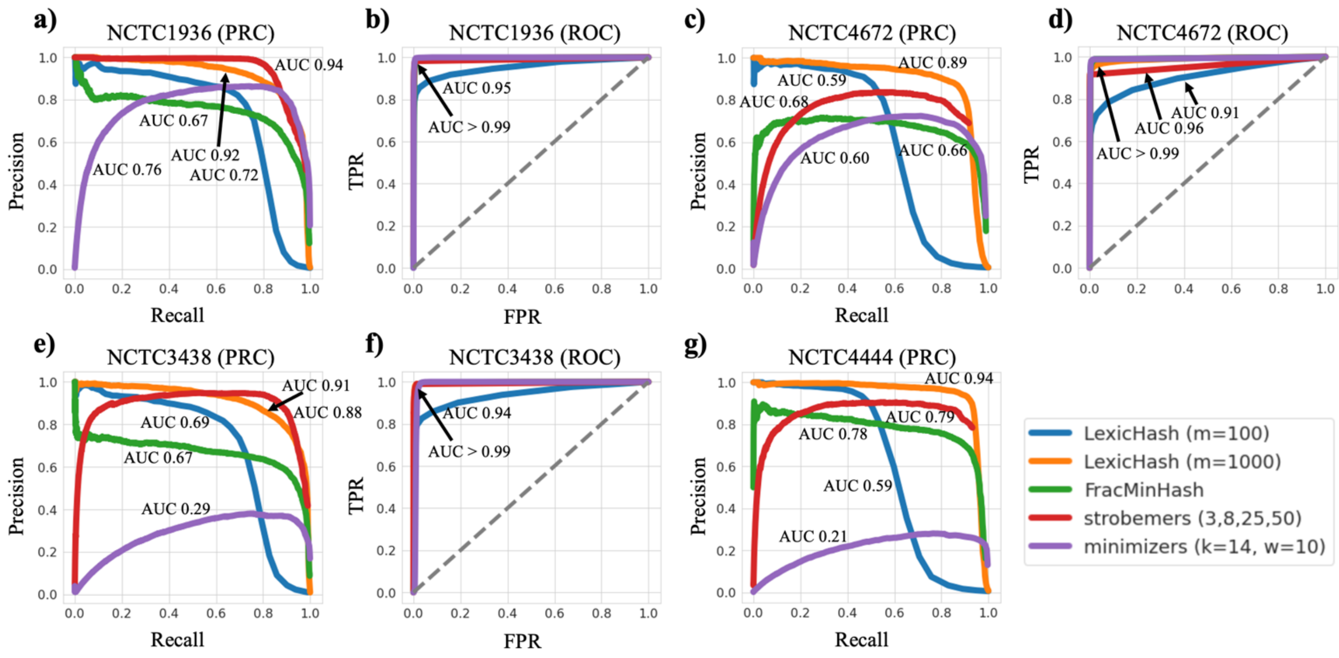

Figure S4. Precision-recall curves (PRCs) and receiver operating characteristic curves (ROCs) for four NCTC datasets using LexicHash with 100 and 1,000 masks, FracMinHash with $m_{eq} = 1000$, strobemers with $(n, k, w_{min}, w_{max}) = (3,8,25,50)$, and minimizers with $(k, w) = (14,10)$. In the majority of NCTC datasets, strobemers outperforms the other methods as in the NCTC1936 (*Klebsiella pneumoniae*) and NCTC3438 (*Avibacterium volantium)* in **(a,b,e,f)**. We also show two examples in **(c,d,g)** where strobemers does not perform as well for the NCTC4672 (*Streptococcus uberis*) and NCTC4444 (*S. enterica)* dataset. Note that the ROC plot for the NCTC4444 dataset is omitted as it is nearly identical to that of NCTC4672.

B. Experiment Details

NCTC Datasets

We used the first 40 NCTC datasets for our experiments. In each dataset we only considered roughly 1000 reads, since our main aim with these datasets was to evaluate the performance of LexicHash, rather than discover all overlaps in a full dataset for practical use. The reads were picked such that each read had at least 5 significant overlaps (according to Daligner), thus allowing us to compare the performance without too many false positives. Daligner was used to generate the ground truths of these alignments for a fixed threshold $\theta = 0.2$. To obtain the ROC curves and PRC curves for MinHash, we considered different thresholds on the calculated Jaccard similarity. For LexicHash we considered different thresholds on the maximum match length between two reads. Disclosure: NCTC datasets were originally preprocessed for the Spectral Jaccard Similarity paper Baharav *et al.* (2020). The above text was modified from the corresponding Supplemental Information.

List of NCTC Datasets Used

NCTC74, NCTC86, NCTC92, NCTC129, NCTC204, NCTC235, NCTC418, NCTC1080, NCTC1936, NCTC2218, NCTC2366, NCTC2669, NCTC3046, NCTC3166, NCTC3168, NCTC3438, NCTC3750, NCTC3761, NCTC4001, NCTC4133, NCTC4136, NCTC4137, NCTC4163, NCTC4168, NCTC4169, NCTC4174, NCTC4199, NCTC4444, NCTC4450, NCTC4669, NCTC4671, NCTC4672, NCTC4673, NCTC4675, NCTC4725, NCTC4840, NCTC5046, NCTC5047, NCTC5050, NCTC5051, NCTC5052

***Plasmodium falciparum* Dataset**

The *P. falciparum* dataset was generated in a similar way as the NCTC datasets. We used minimap2 version 2.17 to find all sets of overlapping read pairs (with mapping quality 60). Then, we pruned the read set to 2000 reads by taking reads with a degree ≥ 40 from the overlap graph. The resulting reads after this process had between 5 and 20 overlaps among the 2000 reads. Minimap2 was used the generate ground truths, and ROCs and PRCs were generated in an identical manner to that of the NCTC datasets.

***Escherichia coli* Dataset**

The E. coli dataset (accession SRR11434956) used to benchmark the runtime comparison between LexicHash and MinHash was sequenced by a PacBio RS II sequencer. The original dataset consisted of 144,238 reads. After preprocessing to exclude reads with > 30% error, the resulting full dataset consists of 113,378 reads. Filtering was done using fastq-filter (https://github.com/LUMC/fastq-filter). Memory and cpu-time were determined using a non-multiprocessed version of LexicHash. The memory usage increases roughly linearly with the number of CPUs used.

Details of minimizer method

The hash function used in the standard minimizer method is identical to that of MinHash (Alg 2 of Section C), using a single hash function for all reads. For chaining, we used 20 bases for the maximum difference in position differences between consecutive collinear hits in a chain (i.e., the ϵ used in Alg 4 of the minimap paper). For example, when computing a chain between sequences s_1 and s_2, suppose a hit occurs at position 200 in s_1 and 400 in s_2. If the next hit occurs at 300 in s_1 and 510 in s_2, then the chain is extended, but not if the hit is at position 600 in s_2, since the difference increases more than 20 (from 200 to 300).

Details of the comparison to strobemers

We note here that when increasing the number of masks used in LexicHash, the performance does not necessarily improve significantly. This is because the basic version of LexicHash simply uses the maximum match length as the similarity score, which can predict spurious matches due to short genomic repeats (of e.g., 40 bp). Thus, to use an increasing number of hash function in the comparison to strobemers and minimizers, we take the average of the top x largest matches as the similarity score, where $x = 2, 3, 4, 8$ for $n_{hash} = 100, 200, 500, 1000$, respectively. This has the effect of lowering the score of these false positive read-pairs, while maintaining large scores for real overlaps.

C. Discussion on mask generation

In this section, we explore the effect that different mask generating processes has on the LexicHash score (i.e., match length). For each dataset and mask type, we evaluate the binary hypothesis test (BHT) between deciding whether a pair of reads is "overlapping" or "nonoverlapping" with equal priors, based on the score for a *single mask*. Particularly, we calculate the probability of error of the BHT,

$$P_e = \pi_{ovlp}\Pr(err \mid ovlp) + \pi_{nonovlp}\Pr(err \mid nonovlp),$$

which happens to be half the area under the minimum of the two overlapping and nonoverlapping PMFs (i.e., the black lines in the figures) since the priors are equal.

The types of mask generation processes evaluated are shown below. In the first experiment, we determine whether generating masks using a deterministic process that evenly "spreads out" the masks is better than randomly generating them by default. In the second, we evaluate whether we can achieve greater statistical power by tailoring the mask generation processes to the dataset at hand. Specifically, we compare generating masks uniformly at random with a distribution that "looks like" the dataset, or like the opposite of the dataset.

Uniform: Masks are generated i.i.d. uniform on {A,T,G,C}. This is the default used in the current version of LexicHash.

Evenly Distributed: Given a choice for the number of masks, m, the starts of the masks use each possible combination of bases, the initial patterns are repeated to a length of $k_{\max}$. For example, if $m = 256 = 4^4$ (which is used to create the plots in Fig S5), the masks are $\overline{AAAA}, \overline{AAAT}, \dots, \overline{GGGG}$.

Biased: Masks are generated i.i.d. with probabilities according to the frequencies of each base in the dataset.

Opposite Biased: Mask are generated according to the reverse-complement of the base frequencies.

The datasets evaluated are:

I.I.D: 10,000 overlapping and 10,000 nonoverlapping read pairs are generated using a i.i.d. distribution with 36% G+C probability, and an 8% probability of substitution error is added for each read.

NCTC1080 and *NCTC3761*: These real datasets have G+C contents of 39.1% and 33.5%, respectively.

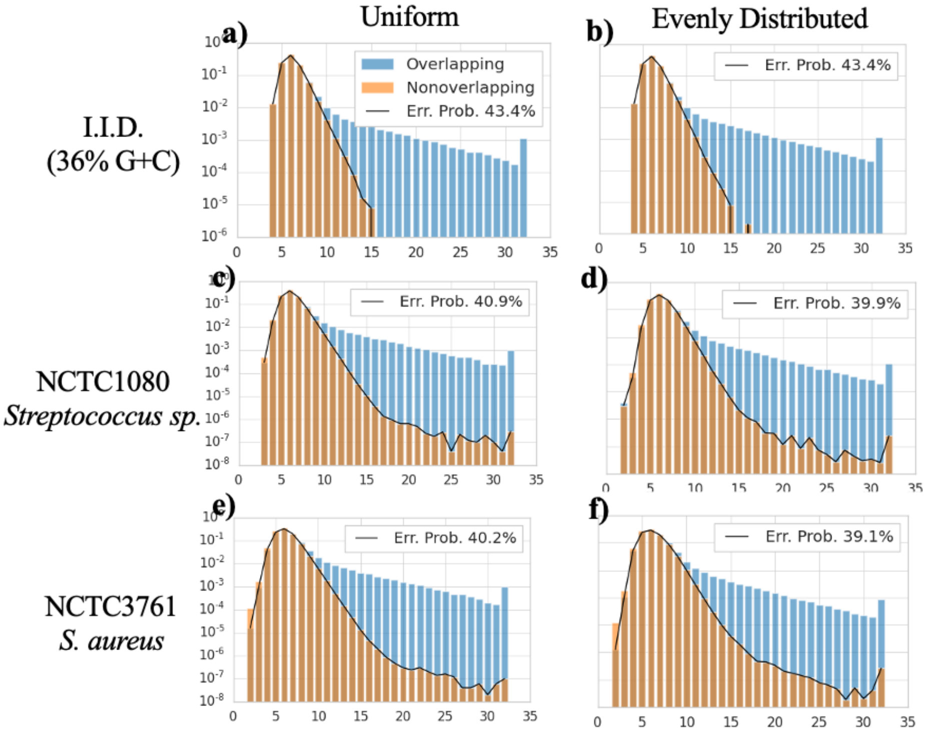

Figure S5. Probability mass functions (PMFs) of the LexicHash score for a single mask, for three different types of mask-generating distributions, across three datasets. For the i.i.d. generated dataset, the difference in error probabilities between the uniform and evenly distributed methods is only 0.1%, which is well within the margin of error. However, the same experiment on the real NCTC datasets shows a significant improvement of around 1% when the masks are generated evenly. This experiment is quite insightful and useful since using a deterministic process to generate the masks decreases the complexity compared to using randomization.

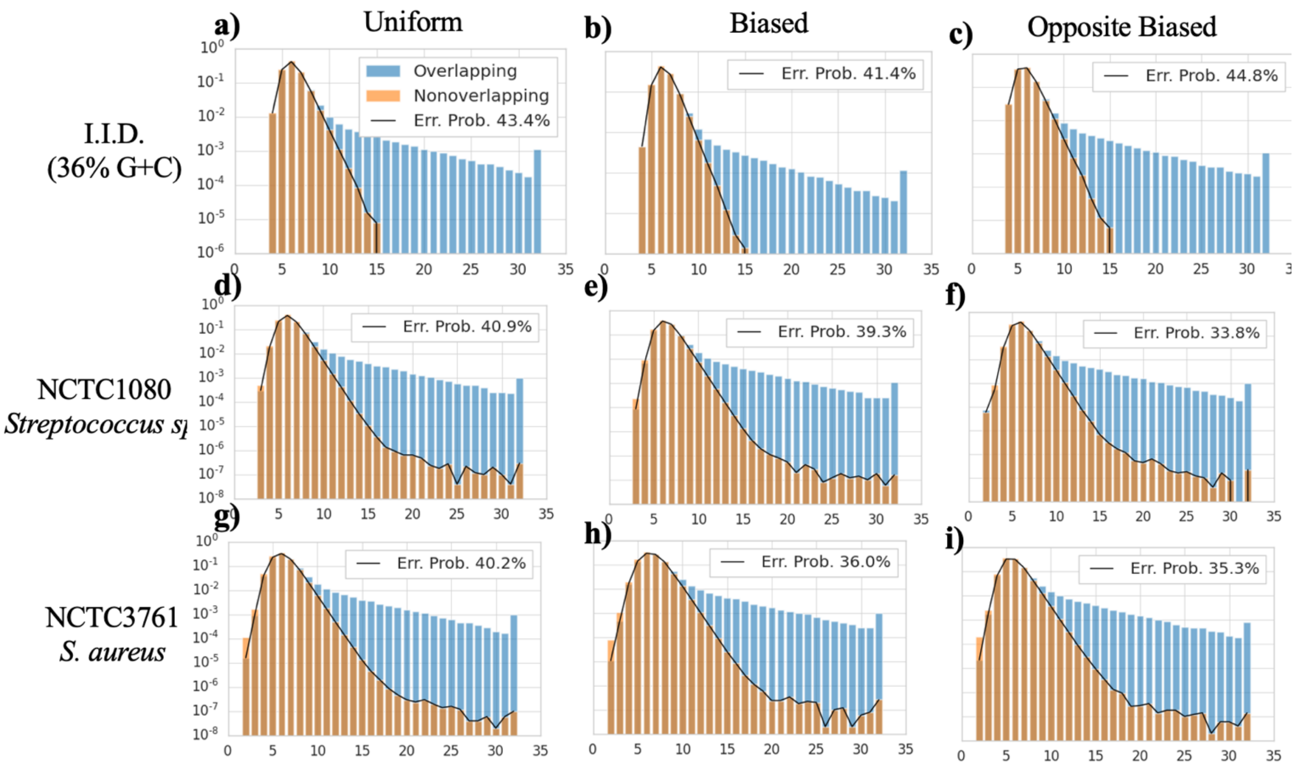

Figure S6. Probability mass functions (PMFs) of the LexicHash score for a single mask, for three types of mask-generating distributions, across three datasets.

Notice that for the i.i.d. dataset, the *biased* mask outperforms the *uniform* mask with a P_e of 41.4%, and the *opposite biased* underperforms. Interestingly for the real NCTC datasets, while the *biased* mask still outperforms the default, the *opposite biased mask* performed the best, which was the case for the majority of the NCTC datasets tested. Clearly, a model with only substitution errors is insufficient to study the role of the generating distribution of LexicHash masks.

Originally, we had hypothesized that the *opposite biased* mask would perform the best of the three, since it would intrinsically “target” less common k-mers, which might theoretically be more important than common ones. Conversely, one possible explanation for why the *biased* mask performs best for the i.i.d. dataset is that it serves as a sort of “matched filter” for detecting k-mers that are likely to be in both sequences for overlapping reads.

D. Additional discussion on runtime and memory

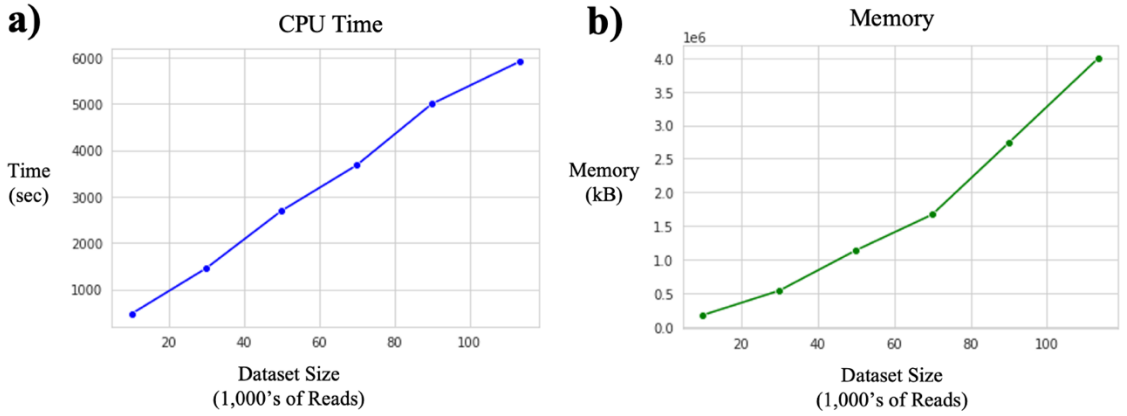

Figure S7. CPU-time and peak memory usage using LexicHash ($k_{max} = 32, k_{min} = 16$) for the *E. coli* dataset with varying sizes after downsampling. The runtime increases roughly linearly with the number of reads due to the efficient similarity estimation implemented in LexicHash. Naturally, the memory usage increases roughly quadratically with the number of reads, since the true number of overlapping read pairs increases quadratically. Note that if the dataset size was further increased, the quadratic scaling would begin to impact the runtime as well if the default LexicHash is used.

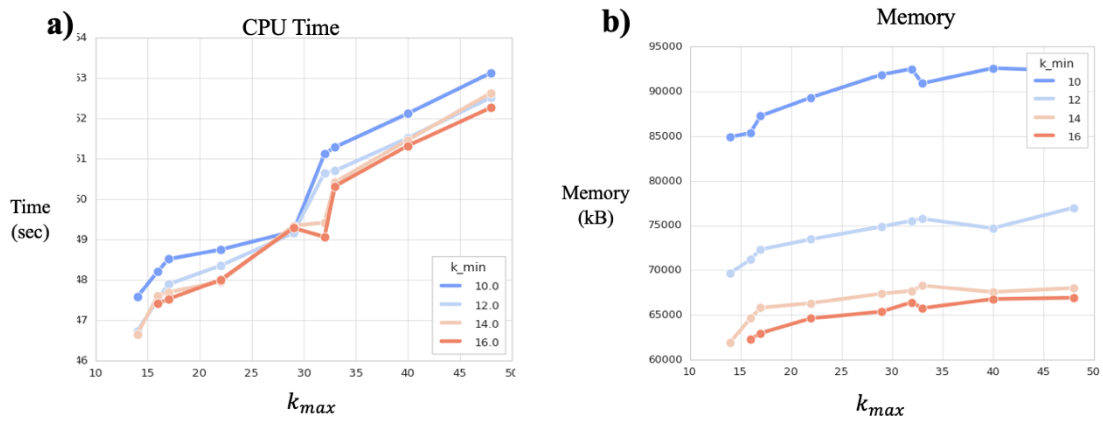

Figure S8. CPU-time and peak memory usage using LexicHash on the NCTC1080 dataset for various k_{max} and k_{min} values. Notice that the memory steadily increases since Python uses more bytes to store larger integers larger (with thresholds at 2^{30} and 2^{60}). Additionally, if LexicHash were implemented in C++, for example, we could expect to see memory and runtime jumps above $k_{max} = 32$, as this corresponds to 64 bits, the typical integer size of modern processors.

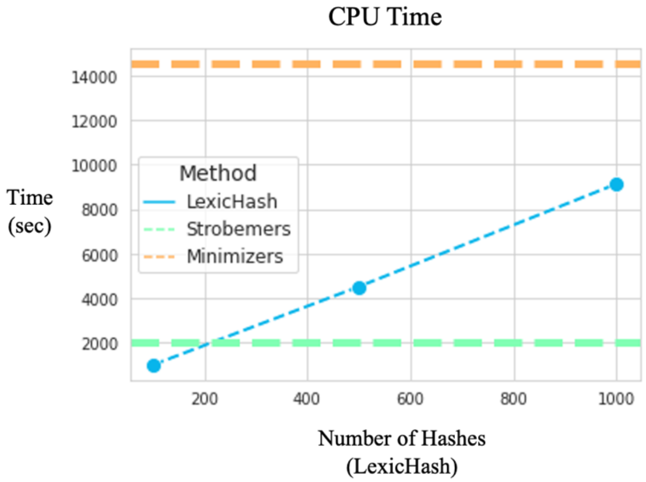

Figure S9. CPU-time for three similarity estimation methods, varying the number of hashes used for LexicHash. We use $(k_{min}, k_{max}) = (16,32)$ for LexicHash, $(n, \ell, w_{min}, w_{max}) = (3,8,25,50)$ for StrobeMap, and $(k, w) = (16,10)$ for minimizers. We can see that the CPU-time increases roughly linearly with the number of hash functions, as expected. As empirically verified in Fig. 8 in the main paper (as well as for other datasets), the performance of LexicHash and strobemers are similar at around 200 hashes, and coincidentally, the CPU-times are also roughly equal at that number of hashes.

E. Algorithms used in the paper

Building the prefix tree

Algorithm 1: Create the prefix tree-like data structure for a given sketch index for all sequences. Each base in the string and mask is converted to an integer a priori, based on the (arbitrary) pre-specified order [A,C,G,T] → [0,1,2,3].

```
Result: Prefix Tree P_i
Input: Min-hashes at Sketch Index i: r_i^(1), ..., r_i^(n), k_max, k_min
begin
   partition ⟵ [[1, ..., n]]
   for k = 1, ... k_max
      next_partition ⟵ []
      for subtree in partition
         for j in subtree
            char ⟵ (r_i^(j) ≫ 2(k_max − k)) AND 11_2
            add j to next_subtree[char]
         for subtree in next_subtree
            if length(subtree) > 1
               add subtree to next_partition
      partition ⟵ next_partition
      if k ≥ k_min
         P_i[k] ⟵ partition
```

Calculating k-mer hash value for MinHash

Algorithm 2: Compute the hash-value for a k-mer for a hash-function specified by coefficients a and b, and hash-size (in number of bits) n_b. In practice, $h_{\max}$ only needs to be computed once for each hash function, but is computed here for instructional purposes.

```
Result: MinHash hash value h
Input: k-mer x, Number of bits n_b, Integer coefficients
         a, b < 4^(n_b)
begin
   h_max ⟵ smallest prime above 4^(n_b)
   h_py ⟵ hash(x)          // uses Python's built-in
    "hash(·)"
   h ⟵ (a × h_py + b) % h_max
```
